# The influence of computer-assisted surgery on rotational, coronal and sagittal alignment in revision total knee arthroplasty

**DOI:** 10.1186/1471-2474-15-94

**Published:** 2014-03-19

**Authors:** Marrigje F Meijer, Martin Stevens, Alexander L Boerboom, Sjoerd K Bulstra, Inge HF Reininga

**Affiliations:** 1 Department of Orthopaedics, University of Groningen, University Medical Center Groningen, P.O. Box 30.001, 9700 Groningen, RB, The Netherlands; 2 Department of Trauma Surgery, University of Groningen, University Medical Center Groningen, P.O. Box 30.001, 9700 Groningen, RB, The Netherlands

## Abstract

**Background:**

Despite good results of primary total knee arthroplasty (TKA), the number of revision total knee arthroplasties (rTKAs) is rising. Proper implant position is essential, since malposition leads to worse clinical outcome. In rTKA most anatomical landmarks have disappeared because of extensive bone loss, making it more difficult to adequately implant the knee prosthesis. In primary TKA, computer-assisted surgery (CAS) leads to better prosthetic alignment than mechanical navigation guides. Literature about the use of CAS in rTKA is scarce though, and the effect on rotational prosthetic alignment has not been investigated yet. Hence the primary objective of this study is to compare rotational prosthetic alignment when using CAS in rTKA compared to a mechanical navigation guide. Secondary objectives are to compare prosthetic alignment in the coronal and sagittal planes. It is hypothesized that CAS leads to better rotational, coronal and sagittal prosthetic alignment when used during rTKA.

**Methods/Design:**

A prospective clinical intervention study with use of a historical control group will be conducted. Forty-four patients with a minimum age of 18 to be admitted for CAS-rTKA between September 2012 and September 2015 will be included in the intervention group. Forty-four patients with a minimum age of 18 who underwent rTKA with the use of a mechanical navigation guide between January 2002 and April 2012 will form the historical control group. Both groups will be matched according to gender and type of revision prosthesis. Rotational prosthesis alignment will be evaluated using a CT-scan of the knee joint.

**Discussion:**

Proper implant position is essential, since malposition leads to worse clinical outcome. Several studies show a significantly positive influence of CAS on prosthetic alignment in primary TKA, but literature about the use of CAS in rTKA is limited. The purpose of this study is thus to investigate the influence of CAS during rTKA on postoperative prosthetic alignment, compared to mechanical navigation guides.

**Trial registration:**

Netherlands National Trial Register NTR3512

## Background

Osteoarthritis (OA) is one of the most prevalent age-related musculoskeletal conditions. Although OA may affect any joint of the body, it is most commonly seen in the hip and knee [1]. OA of the knee leads to a significant impairment in patients’ ability to perform activities of daily living and has a large impact on health-related quality of life [2,3]. For advanced OA of the knee, total knee arthroplasty (TKA) is a highly successful and widely applied surgical treatment, with 450,000 primary TKAs performed in the United States in 2005 [4] and 21,475 TKAs in the Netherlands in 2010 [5]. Due to a growing elderly population and changing thresholds for surgery, these numbers are expected to increase dramatically in the coming decades [4,6].

As a result, the number of TKA revisions (rTKA) will also increase. The demand for rTKA is expected to double by 2015 and a growth of 601% is predicted for the United States between 2005 and 2030 [7]. A similar trend is expected for other Western countries. Main reason for rTKA is aseptic loosening, which accounts for 30-42% of all rTKAs. Infection is the second most common indication and is responsible for about 20% of all revisions. Other reasons for rTKA may be pain, instability, wear, fracture, malalignment, implant breakage, incorrect size and dislocation of one or more components [8,9]. Main reasons for rerevision after rTKA are infection (35-46%), followed by aseptic loosening (19-30%). Other reasons for rerevision are wear/osteolysis, instability, stiffness and periprosthetic fractures [10,11].

The goal of both primary and revision TKA is to restore function and stability of the knee joint and to alleviate pain. However, rTKA is a more complicated surgical procedure than primary TKA and leads to worse clinical results. Major differences between revision and primary TKA are the amount of bone loss and ligamental damage [12,13]. Reasons for this are osteolytic lesions caused by wear, aseptic loosening or infection, and removal of the primary implant.

Proper positioning of the implant is important, since malpositioning of a knee prosthesis leads to worse patient outcome and wear of the prosthesis [14]. Optimal prosthetic alignment is therefore an essential part of the surgical procedure. In primary TKA, one can identify anatomical landmarks and use them to determine the position of the implant using mechanical navigation guides. However, in rTKA most of the time anatomical landmarks have disappeared because of extensive bone loss, making it more difficult to adequately implant the prosthesis.

Several computer navigation systems have been developed to improve prosthetic alignment. In primary TKA, computer-assisted surgery (CAS) is shown to lead to better prosthetic alignment than mechanical alignment guides [15-23]. Several studies have shown improved postoperative mechanical axis as well as coronal, sagittal and rotational prosthetic alignment when using CAS during primary TKA [15,16,24]. Perlick et al. [25] revealed a significantly better mechanical limb axis and coronal alignment of the femoral component when CAS was used during rTKA. However, literature about the use of CAS in rTKA is scarce [25,26] and potential differences in rotational alignment of the prosthesis have not yet been investigated. It is hypothesized that CAS also results in a more accurate prosthetic alignment when used in rTKA. Correct alignment of the prosthesis is more difficult during rTKA compared to primary TKA because of extensive bone loss and the disappearance of anatomical landmarks. This may imply that one can expect more advantages from CAS in rTKA than from primary TKA.

Hence the primary objective of this study was to investigate the effect of CAS on rotational prosthetic alignment when used in rTKA. The effect of CAS on prosthetic alignment in the coronal and sagittal planes will also be determined.

## Methods/Design

### Study design

A prospective clinical intervention study with use of a historical control group will be conducted. In the prospective intervention group patients will undergo rTKA using CAS. These surgeries will take place between September 2012 and September 2015. The historical control group will consist of patients who underwent rTKA between January 2002 and April 2012. The intervention and control groups will be matched according to gender and type of revision prosthesis. The study design, procedures and informed consent are approved by the Medical Ethics Committee of University Medical Center Groningen (UMCG).

### Study population

The study will be conducted at the Orthopedic Department of UMCG. Inclusion criteria for the intervention group are:

– Use of CAS during rTKA.

– Minimum age of 18 years.

– Total revisions, re-implantations and partial revisions of either the tibial or the femoral part are included. For partial revisions, only measurements of the part of the prosthesis to be revised will be used.

Inclusion criteria for the historical control group are:

– rTKA without the use of CAS.

– Minimum age of 18 years.

– Total revisions, re-implantations and partial revisions of either the tibial or the femoral part are included. For partial revisions, only measurements of the part of the prosthesis that was revised will be used.

– Patients will be included if the NexGen® revision system (Zimmer Inc., Warsaw, Indiana, USA) was used.

Exclusion criteria for both groups are:

– Insert replacements and placement of a patellar button only.

– Patients who receive a tumor prosthesis during rTKA.

– Patients with a limited knowledge of the Dutch language or who are mentally incapable of participating.

In both the intervention group and the historical control group the anesthetic, analgesic and postoperative physiotherapy protocols are identical.

### Surgical procedure rTKA

rTKA can be described in three steps: 1) removal of implant, 2) classification of defects and 3) rebuilding of joint by “tibia first technique”. The first step is to extract the failing components and to remove all debris to create a new situation. Hereby bone will be preserved as much as possible, although bone of poor quality has to be removed.

The second step is to classify the bone defects according to the Anderson Orthopaedic Research Institute (AORI) [27]. In defect types 2a, 2b and 3 bone loss will be compensated by metal augmentations, while stems attached to the tibial and femoral components will spread the load to the implant interfaces to secure fixation.

The third step is the rebuilding of the knee joint with a revision prosthesis. The NexGen® revision system (Zimmer Inc., Warsaw, Indiana, USA), is used at the Department of Orthopedics of UMCG. Depending on the bone defects the NexGen Legacy Condylar Constraint Knee® (LCCK) (type 2a or 2b) or the NexGen Rotating Hinge Knee® (RHK) (type 3) is used. Tibial and femoral revision components are placed with press-fit stems, and if needed with augmentation blocks or trabecular metal cones. The revision prosthesis is fixed with bone cement (Refobacin® revision bone cement with clindamycin and gentamicin, Biomet Inc., Warsaw, Indiana, USA). Depending on the stability and integrity of the collateral ligaments, the type of articulating surface is chosen during surgery. With good collateral ligaments a posterior stabilizing component (Legacy Posterior Stabilized®, LPS) will give sufficient stability. However, in case of coronal plane instability a semi-constraint insert (LCCK) with a high post is needed. With gross collateral deficiency and multidirectional instability a rotational hinge is the best choice (RHK).

### Intervention group

In the intervention group, CAS will be applied during rTKA. The ORTHOsoft Navitrack® navigation system (Zimmer Inc., Warsaw, Indiana, USA) will be used. The navigation is based on an infrared reflecting system with use of trackers in the femur and tibia. The system guides the surgery by an image-free model based on anatomical landmarks identified by the surgeon. After the exposure a femoral tracker is placed proximally from the knee in the same knee wound or in an additional 3-cm incision. The tibial tracker will be placed in an additional 3-cm incision above the ankle. Before removal of the primary prosthesis, the navigation protocol is applied in which the anatomical landmarks are chosen and the system will build its model from the patient’s data. Thus, all anatomical landmarks are identified with the primary prosthesis in situ. The mechanical axis of the lower limb as measured with this system is the angle between the mechanical axis of the femur and tibia. The mechanical axis of the femur is the axis between the center of the femoral head and the deepest point of the intercondylar notch. The center of the femoral head is determined by moving the leg in a conical pattern, digitizing 14 distinct positions of the femoral tracker. The deepest point of the intercondylar notch is marked by the orthopedic surgeon. The mechanical axis of the tibia is the axis between the entry point of the proximal medullary canal and the center of the ankle. The entry point of the medullary canal is marked by the orthopaedic surgeon and the center of the ankle is assessed by marking the medial and lateral malleoli. Coronal and sagittal prosthesis alignment of the femoral and tibial components are calculated according to respectively the femoral and tibial mechanical axis. Rotation of the femoral component is determined according to the epicondylar axis. The orthopaedic surgeon marks the medial and lateral epicondyle and thus this axis is generated. Rotation of the tibial component is assessed in relation to the axis between the middle of the posterior cruciate ligament insertion and the medial third of the tibial tuberosity. Both landmarks are marked by the orthopaedic surgeon. The navigation system will guide the surgery in positioning the components and choosing the size of the implants.

When implanting a press-fit stem, it may be that alignment of the components are influenced by the stem. After removal of the primary prosthesis and preparation of the bone cuts, different provisional components are tried in order to determine the correct type and size of the components. In this way, and by checking the alignment of the components with the navigation system, the orthopaedic surgeon will know if the stem influences the component alignment. In the revision system we have the availability of straight stems and off-set stems to use the best position of the component in combination with the stem but not forced by the stem. When this is the case, a stem of a smaller diameter will be chosen, so that the components are placed according to the bone cut made using the navigation system instead of the stem. Cementing underneath the tibial tray and leaving the stem uncemented generally provides enough component stability. In the rare case of the components not being rotationally stable, the stem will be cemented.

### Historical control group

In the control group the position of the revision prosthesis was determined using mechanical intramedullary alignment guides for the femur and tibia. Positioning of the components and sizing were based on the same anatomical landmarks as in the intervention group with use of prototypes and trial components.

### Study procedures

Demographic characteristics, BMI, indication for operation, type and brand of prosthesis, total amount of blood loss, surgical time, length of hospital stay and ASA classification will be collected and/or recorded for all patients included in this study.

### Radiographic evaluation

Rotational prosthetic alignment will be measured on a CT-scan of the operated leg. For evaluation of alignment in the coronal and sagittal planes a new imaging device, called the EOS system (Biospace Imaging, Paris, France) [28], will be used. This device is characterized by a reduction in radiation (800-1000 times less than for CT-scan and 10 times less than conventional X-ray) [28,29]. Reason for this is the use of an innovative technology called fast gaseous particle detectors, invented by George Charpak (which earned him the Nobel prize in physics in 1992). The EOS imaging device uses two orthogonal sources of radiation and linear detectors that are coupled together. These sources move up and down along the patient, producing an anterior-posterior and lateral image at the same time while the patient is in weightbearing position. This is a different technique than conventional radiograph systems, where beams are divergent in horizontal and vertical plane.

Of the patients in the intervention group, a CT-scan of the operated leg will be made postoperatively during the visits at the outpatient clinic. Patients included in the historical control group have already undergone the standard surgical technique for rTKA. A CT-scan of the operated leg will be made the next time the patient visits the outpatient clinic of the Orthopedic Department for follow-up of the rTKA.

For evaluation of rotational prosthetic alignment, rotation of the femoral and tibial components will be determined separately according to the Berger CT protocol [30]. Angles measured for rotational alignment are:

– Condylar twist angle for rotation of the femoral component: angle between the epicondylar axis and the prosthetic posterior condylar axis (inner border of posterior cut). Endorotation of the femoral component will be shown as a positive (+) angle and exorotation of the femoral component will be shown as a negative (-) angle. An angle of >3° endorotation or exorotation will be considered an outlier.

– Rotation of the tibial component: angle between the tibial tubercle axis (axis between the geometric center of the proximal tibial plateau and the tip of the tubercle) and the tibial component angle (anterior-posterior line through the tibial component). Normal rotation of the tibial component is considered 18° endorotation [30]. Endorotation of the tibial component will be shown as a positive (+) angle and exorotation of the tibial component will be shown as a negative (-) angle. An angle of >3° endorotation or exorotation will be considered an outlier.

Prosthetic alignment in the coronal and sagittal planes will be measured using postoperative coronal and sagittal X-rays of the operated leg. These lower-limb X-rays are obtained using the EOS system (Biospace Imaging, Paris, France) [28] EOS 2D images will be used for measuring alignment. For the intervention group, standard coronal and sagittal X-rays will be taken postoperatively as part of the standard operation protocol. For the historical control group, standard coronal and sagittal X-rays have already been taken postoperatively.

Angles measured for coronal and sagittal alignment are:

– Mechanical angle of the leg (HKA): Angle between the line from the femoral head to the center of the knee and the line from the center of the ankle to the center of the knee in coronal plane.

– Mechanical lateral distal-femoral angle (mLDFA): Angle between the mechanical axis of the femur and the articular surface of the femoral part of the prosthesis in coronal plane.

– Mechanical medial proximal tibial angle (mMPTA): Angle between the mechanical axis of the tibia and the articular surface of the tibial part of the prosthesis in coronal plane.

– Anatomical proximal posterior tibial angle (aPPTA): Angle between the mechanical axis of the tibia and the articular surface of the tibial part of the prosthesis in sagittal plane. Downslope of the design of Nexgen prostheses is 7°, and this angle is considered the normal aPPTA.

– For the mechanical axis of the leg, mLDFA, mMPTA and aPPTA a generally accepted outlier cut-off of +/-3° will be applied in this study [31-34].

The canal-filling ratio (CFR) will be determined in both the intervention and control groups to assess whether the stems are canal-filling. The CFR will be measured on the coronal and sagittal EOS images as described by Parsley et al. [35]. The stem diameter and endosteal diameter will be measured at the stem tip. The CFR will be calculated by dividing the stem diameter by the endosteal diameter. A stem is considered to be canal-filling when the CFR is ≥0.85 [35].

### Sample size

The hypothesis is that the use of CAS leads to fewer outliers in prosthetic alignment compared to the use of mechanical alignment guides during rTKA. Primary outcome measure will be rotational prosthetic alignment. When using the conventional operation technique, around 25% of the knees is considered a radiological outlier. Therefore, a P2 value of 0.75 was chosen [21,36]. Previous research has shown that the use of CAS in TKA decreases the number of outliers by 17-30% [21,37,38]. When a 20% decrease in outliers is expected in the CAS group compared to the control group with P1 = 0.95, P2 = 0.75, power = 80% and alpha = 0.05, 44 patients per group are needed.

Since 2008 a total of amount of 21-30 rTKAs have been performed each year at UMCG, and an increase is expected. As a result of that, the possibility is expected of including 44 patients in the intervention group between September 2012 and September 2015. Inclusion of 44 patients in the historical control group is not expected to be a problem, as 165 rTKAs were performed between January 2002 and April 2012.

### Statistical analysis

All statistical analyses will be performed using the PASW software package (version 19, SPSS, Chicago, USA). Descriptive statistics will be used to describe the main characteristics of both research groups. Differences in rotational, coronal and sagittal alignment between the groups will be determined by using the nonparametric Mann-Whitney U-test for independent samples. For the clinical parameters, t-tests will be used for continuous values or the Mann-Whitney U-test when the variables are not normally distributed. A Chi-square test and a Fisher’s Exact test will be used for dichotomous values. For all test procedures, a p-value of < .05 will be considered to indicate statistical significance.

## Discussion

Correct prosthetic alignment is important for a good clinical outcome after TKA. More accurate alignment after TKA correlates with less pain, better knee function, faster rehabilitation and improved quality of life [39,40]. Rotational malalignment has a negative effect on patellar tracking, stability, pain and overall biomechanics of the knee joint [30,41-44], while malalignment in the coronal and sagittal planes leads to an increased risk of loosening, pain and instability [31,34,45,46]. Accurate prosthetic alignment is therefore essential during primary and revision TKA, to postpone revision and rerevision procedures.

In recent years, CAS has become a frequently used technique for improving prosthetic alignment in primary TKA, and several studies have shown its benefits. CAS significantly improves varus/valgus angle [15,17-20,47], mLDFA [16,17,21-23], mMPTA [16,17,20,21,23], femoral flexion angle [15,16,18,21,22], tibial downslope [15,18,20,22,23] and rotational alignment for the femoral and tibial components [15,16,20]. Perlick et al. [25] revealed a significantly better mechanical limb axis and coronal alignment of the femoral component when CAS was used during rTKA. Correct alignment of the prosthesis is even more difficult during rTKA compared to primary TKA because of extensive bone loss and the disappearance of anatomical landmarks. This may imply the expectation of an even greater advantage of CAS in rTKA compared to primary TKA. However, literature about the use of CAS in rTKA is scarce [25,26,48,49]. Moreover, patient groups in these studies are small and only one study has compared postoperative prosthetic alignment with a control group. Furthermore, potential differences in rotational alignment of the prosthesis have not yet been investigated.

In conclusion, it is our expectation that this study will provide insight into the effectiveness of CAS in rTKA on postoperative prosthetic alignment. It is our hypothesis that the use of CAS in rTKA leads to improved prosthetic alignment compared to conventional rTKA.

## Competing interests

A.L. Boerboom is a paid consultant for Zimmer GmbH who does training in knee arthroplasty and computer-assisted surgery. The Department of Orthopedics of UMCG receives institutional research support from Zimmer GmbH. All other authors declare that they have no competing interests.

## Authors’ contributions

ALB and SKB originated the idea for the study, led its design, performed and will perform the revision total knee arthroplasties, and will supervise the project. MS, IHFR, ALB and SKB participated in the design of the study and research protocols. IHFR will provide statistical consultation. MM will coordinate the study, is responsible for data acquisition and will conduct the statistical analyses. All authors read and approved the final manuscript.

## Pre-publication history

The pre-publication history for this paper can be accessed here:

http://www.biomedcentral.com/1471-2474/15/94/prepub
